# Molecular underpinnings of neurodegenerative disorders: striatal-enriched protein tyrosine phosphatase signaling and synaptic plasticity

**DOI:** 10.12688/f1000research.8571.1

**Published:** 2016-12-29

**Authors:** Paul J. Lombroso, Marilee Ogren, Pradeep Kurup, Angus C. Nairn

**Affiliations:** 1 Child Study Center, Yale University School of Medicine, New Haven, Connecticut, 06520, USA; 2 Department of Psychiatry, Yale University School of Medicine, New Haven, Connecticut, 06520, USA; 3 Department of Neuroscience, Yale University School of Medicine, New Haven, Connecticut, 06520, USA; 4 Department of Psychology, Boston College, Chestnut Hill, MA, 02467, USA

**Keywords:** alzheimer's disease, parkinson's disease, striatal-enriched protein tyrosine phosphatase, synaptic plasticity, neurodegenerative disorder

## Abstract

This commentary focuses on potential molecular mechanisms related to the dysfunctional synaptic plasticity that is associated with neurodegenerative disorders such as Alzheimer’s disease and Parkinson’s disease. Specifically, we focus on the role of striatal-enriched protein tyrosine phosphatase (STEP) in modulating synaptic function in these illnesses. STEP affects neuronal communication by opposing synaptic strengthening and does so by dephosphorylating several key substrates known to control synaptic signaling and plasticity. STEP levels are elevated in brains from patients with Alzheimer’s and Parkinson’s disease. Studies in model systems have found that high levels of STEP result in internalization of glutamate receptors as well as inactivation of ERK1/2, Fyn, Pyk2, and other STEP substrates necessary for the development of synaptic strengthening. We discuss the search for inhibitors of STEP activity that may offer potential treatments for neurocognitive disorders that are characterized by increased STEP activity. Future studies are needed to examine the mechanisms of differential and region-specific changes in STEP expression pattern, as such knowledge could lead to targeted therapies for disorders involving disrupted STEP activity.

## Introduction

Protein tyrosine phosphorylation is regulated by the fine balance of the activity of protein tyrosine kinases and protein tyrosine phosphatases (PTPs) and plays a critical role in many cellular activities, including gene regulation, cell growth, differentiation, migration, and synaptic plasticity ^1^. Enormous progress in the understanding of PTP function followed the biochemical purification and characterization of the first PTP (PTP1B) over 30 years ago ^2, 3^. Moreover, dysregulation or mutations in genes that encode PTPs lead to metabolic, neurological, developmental, and psychiatric disorders ^1, 4– 6^. These important advances have motivated efforts to find PTP inhibitors that are effective against diabetes, cancer, neurodegeneration, and other serious disorders ^7, 8^.

This commentary focuses on striatal-enriched PTP (STEP), which is found in the central nervous system (CNS), and how increased STEP activity contributes to several disorders, including Alzheimer’s disease (AD) and Parkinson’s disease (PD). In particular, we focus on the ways in which modulating STEP activity contributes to impaired neuronal communication.

STEP affects neuronal communication by opposing synaptic strengthening through the coordinated dephosphorylation of multiple substrates that regulate synaptic plasticity. As discussed in detail below, these substrates include subunits of both the N-methyl-D-aspartate receptors (NMDARs) and the α-amino-3-hydroxy-5-methyl-4-isoxazolepropionic acid receptors (AMPARs). Tyrosine dephosphorylation of these receptor subunits leads to internalization of NMDAR or AMPAR complexes, which diminishes synaptic strength ^9– 13^. High levels of STEP in human brain tissue from AD or PD subjects, as well as animal models of AD and PD, are believed to disrupt synaptic function and to contribute to the learning deficits present in these disorders. These findings are consistent with the growing interest in synaptopathology, or the hypothesis that disorders of cognitive function involve disrupted synaptic function, at least in the earliest stages of neurodegenerative disorders ^14, 15^. This commentary focuses on AD and PD. The role of STEP in other CNS disorders is discussed in several recent reviews ^4, 6, 16^.

## The functional importance of STEP structure

**Figure 1.**
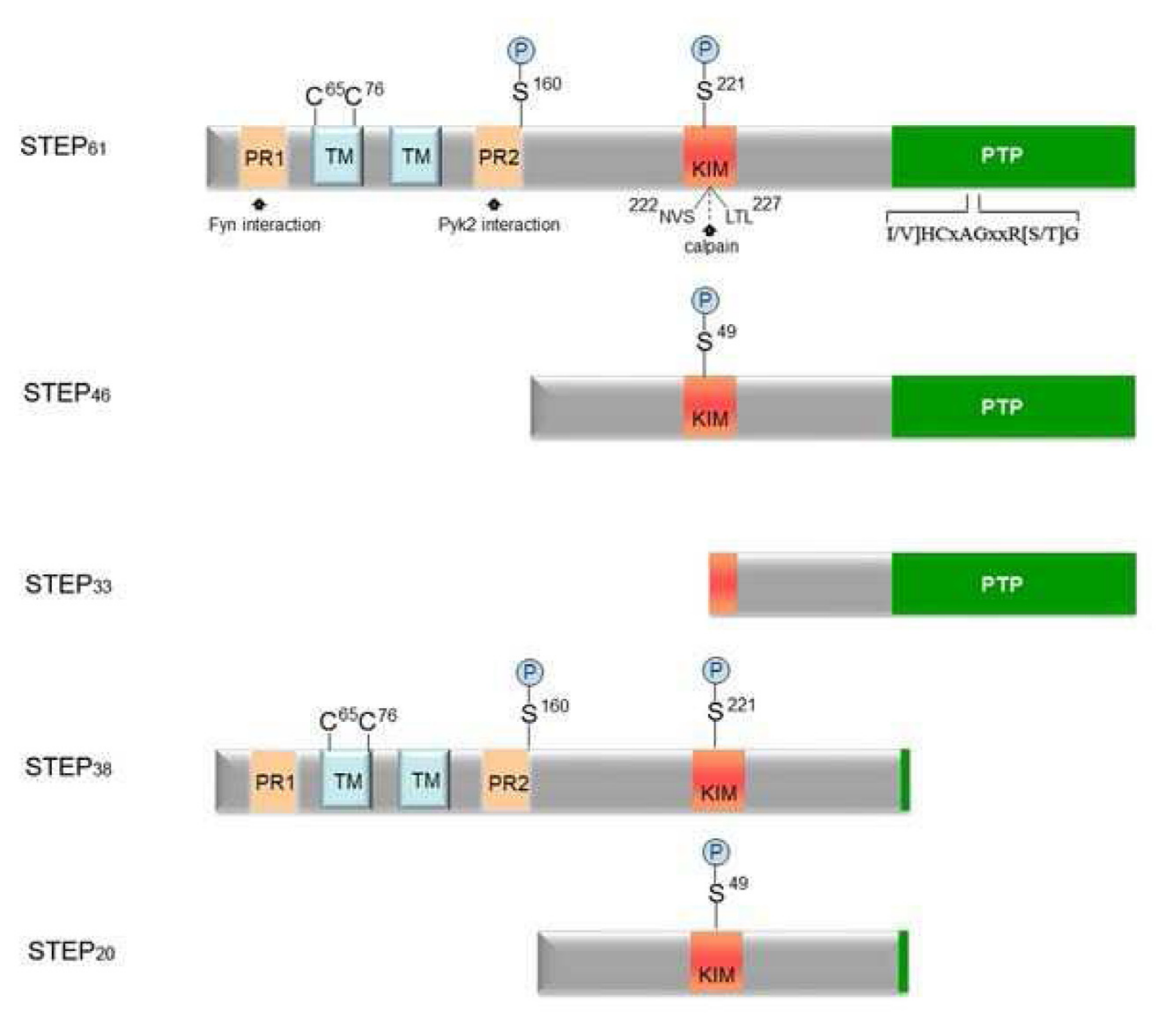
Regulatory domains present in striatal-enriched protein tyrosine phosphatase (STEP). Four isoforms of STEP (STEP _61_, STEP _46_, STEP _38_, and STEP _20_) are produced by alternative splicing of a single STEP gene ( *PTPN5*). Calpain cleavage produces an additional form of STEP (STEP _33_). STEP _61_ and STEP _46_ are the major STEP proteins in the central nervous system (CNS). The kinase-interacting motif (KIM) domain is necessary for interaction with substrates, and the consensus protein tyrosine phosphatase (PTP) sequence, [I/V]HCxAGxxR[S/T]G, is required for phosphatase activity. STEP _38_ and STEP _20_ do not contain the PTP sequence and are inactive variants of STEP with unknown function. STEP _33_ is generated by calpain cleavage within the KIM domain between Ser ^224^ and Leu ^225^. Cleavage at this site disrupts the ability of STEP _33_ to bind to substrates. STEP _61_ contains an additional 172 amino acids at the N-terminus that possesses two transmembrane (TM) domains and two polyproline-rich (PP) regions. The TM regions target STEP _61_ to the endoplasmic reticulum and to synaptic and extrasynaptic sites. While the KIM domain is required for binding to STEP substrates, the PP regions impart some degree of substrate specificity, with Fyn binding to PR1 and Pyk2 binding to PR2. PKA phosphorylates STEP within the KIM domain (Ser ^221^ and Ser ^49^ on STEP _61_ and STEP _46_, respectively), as well as in the region adjacent to the PP regions (Ser ^160^ on STEP _61_). The function of Ser ^160^ phosphorylation of STEP _61_ remains unclear. Finally, two cysteine residues, Cys ^65^ and Cys ^76^, present within the TM region promote dimerization of STEP and reduce its phosphatase activity ^93^.

STEP, encoded by the *PTPN5* gene, is highly expressed throughout the CNS, with the exception of the cerebellum ^17– 21^. STEP is alternatively spliced to produce four related proteins ( Figure 1), with the most abundant isoforms being STEP _61_ and STEP _46_^22, 23^. STEP _61_ associates with membrane compartments using a unique 172-amino-acid domain at its N-terminus that is not present in STEP _46_. This domain contains two hydrophobic regions that target STEP _61_ to the endoplasmic reticulum (ER) and synaptic as well as extrasynaptic membranes. In contrast, STEP _46_ is a cytosolic protein ^19, 24, 25^. Both STEP _61_ and STEP _46_ contain, at their C-terminus, the consensus PTP sequence ([I/V]HCxAGxxR[S/T]G) that is required for catalytic function. Upstream of the catalytic domain is a kinase-interacting motif (KIM), the substrate-binding domain necessary for associating STEP with all known substrates ^25– 27^.

## STEP substrates: glutamate receptors

STEP regulates the trafficking of two glutamate receptor subtypes, NMDARs and AMPARs ^9, 10, 13, 28– 30^. NMDARs are internalized after GluN2B dephosphorylation (at Tyr ^1472^), which facilitates the binding of GluN2B to clathrin adaptor proteins and promotes the internalization of GluN1/GluN2B receptor complexes ^31^. Consistent with this finding, STEP knockout mice display increased synaptosomal GluN1/GluN2B receptors and increased NMDAR excitatory post-synaptic currents, which appears to facilitate hippocampal and amygdala learning ^29, 30, 32, 33^. Similarly, GluA2 dephosphorylation promotes internalization of GluA1/GluA2 receptor complexes; whether internalized NMDAR and AMPAR complexes are recycled or degraded is not yet known.

Internalization of GluA1/GluA2 AMPARs results from tyrosine dephosphorylation of the GluA2 subunit ^34, 35^. STEP is the PTP that mediates this process ^12, 36^. Stimulating mGluRs with the agonist DHPG (S-3,5-dihydroxyphenylglycine) leads to internalization of GluA1/GluA2 ^12^. DHPG stimulation of mGluRs increases the local translation of STEP, resulting in the subsequent dephosphorylation and endocytosis of GluA1/GluA2. Moreover, neuronal cultures from STEP knockout mice display increased surface expression of AMPARs and do not undergo DHPG-mediated AMPAR endocytosis; however, internalization of AMPARs can be restored with the re-introduction of STEP into the knockout mouse cultures ^12^.

## STEP substrates: other synaptic substrates

Additional STEP substrates involved in synaptic strengthening include two members of the mitogen-activated protein kinase (MAPK) family, extracellular signal-regulated kinases 1 and 2 (ERK1/2), and p38 ^37– 43^. STEP dephosphorylates regulatory tyrosine residues within their activation loops and thereby inactivates them. ERK1/2 and p38 have opposing actions: ERK1/2 promotes synaptic strengthening and p38 promotes cell death pathways. STEP and both MAPKs reside in dendritic spines, raising the question of how STEP regulates the activity of two proteins with such different cellular actions ^43^. The balance between the activation of synaptic or extrasynaptic NMDARs appears to be critical to this regulation ^44, 45^.

**Figure 2.**
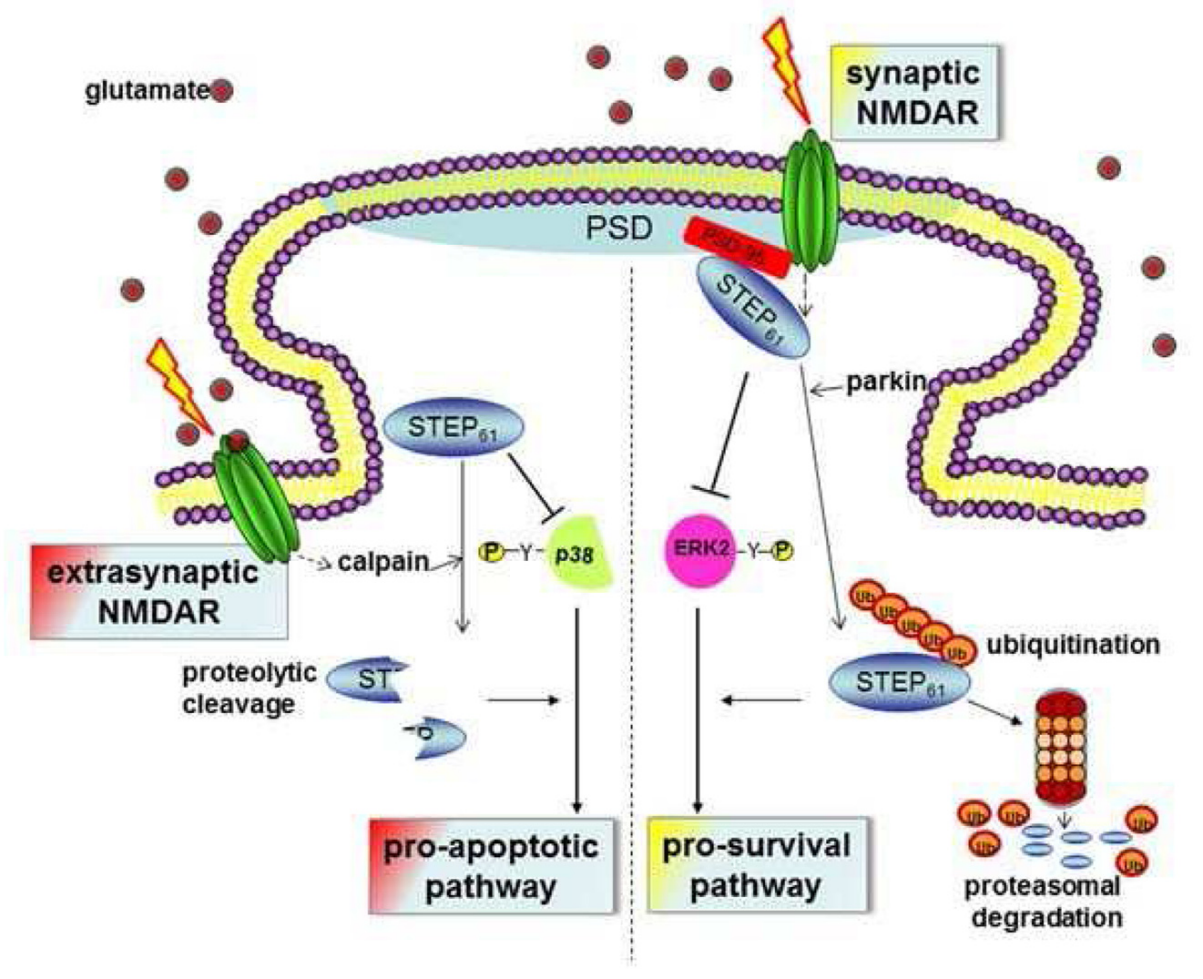
Differential regulation of ERK and p38 by synaptic versus extrasynaptic stimulation. Extrasynaptic N-methyl-D-aspartate receptor (NMDAR) stimulation invokes calpain-mediated proteolysis of striatal-enriched protein tyrosine phosphatase 61 (STEP _61_), producing a truncated cleavage product, STEP _33_. STEP _33_ is unable to bind to and dephosphorylate its substrates. The stress-activated mitogen-activated protein kinase (MAPK) p38 is preferentially activated by extrasynaptic NMDAR stimulation, and cell death pathways are subsequently initiated. Cleavage of STEP _61_ is therefore likely a component of excitotoxic insults associated with stroke/ischemia and Huntington’s disease. On the other hand, synaptic NMDAR stimulation leads to the activation of multiple kinases responsible for phosphorylating STEP _61_ and recruiting the ubiquitin proteasome system to dendritic spines. Post-synaptic density (PSD) protein 95 (PSD-95) binds to STEP _61_ through its third PDZ domain, and the binding of PSD-95 to STEP _61_ promotes the rapid ubiquitination and degradation of STEP _61_ by the proteasome ^46^. As PSD-95 stabilizes NMDARs within the PSD, removing the negative regulator STEP promotes synaptic strengthening. Synaptic NMDAR stimulation results in the degradation of STEP _61_, leads to an increase in ERK1/2 activation, and promotes neuronal survival.

Synaptic stimulation leads to STEP ubiquitination and consequent degradation and to the activation of ERK1/2 but not p38 ( Figure 2). At synaptic sites, STEP _61_ binds to post-synaptic density protein 95 (PSD-95) but not to other PSD-95 family members, and the binding of PSD-95 to STEP _61_ promotes rapid STEP _61_ ubiquitination and then degradation by the proteasome ^46^. As PSD-95 stabilizes NMDARs at the postsynaptic density, removing the negative regulator STEP promotes synaptic strengthening. Extrasynaptic sites display a two- to three-fold increase in STEP _61_ levels compared to synaptic sites ^47^. As glutamate levels increase at the synapse, extrasynaptic NMDARs are engaged and calcium influx activates calpain and STEP _61 _cleavage ( Figure 2). The cleavage occurs in the substrate-binding KIM domain, releasing a smaller STEP variant (STEP _33_) that no longer binds to or dephosphorylates STEP substrates. In contrast to the stimulation of synaptic NMDARs, the stimulation of extrasynaptic NMDARs activates p38 and downstream cell death signaling pathways, but not ERK1/2. In support of this model of STEP function, the addition of a STEP-derived peptide that spans the calpain cleavage site competitively blocks proteolysis and neurons are protected from glutamate-mediated excitotoxicity ^44^.

Two other STEP substrates are Pyk2 and Fyn, where dephosphorylation of the regulatory tyrosines in their activation loops inactivates these kinases ^48, 49^. STEP _61_ has two polyproline-rich regions that, in addition to the KIM domain, are involved in substrate binding and contribute to substrate specificity; the first polyproline domain facilitates binding to Fyn ^48^, while the second polyproline domain is necessary for binding to Pyk2 ^49^ ( Figure 1). Of note, Fyn phosphorylates GluN2B at Tyr ^1472^, the same site that is dephosphorylated by STEP. Thus, STEP dephosphorylates GluN2B directly and at the same time dephosphorylates and inactivates the kinase that phosphorylates GluN2B ^10, 48, 50^.

The most recently identified STEP substrate is PTP alpha, an activator of Fyn ^51^. In contrast to STEP, which dephosphorylates the activation loop and thereby inactivates Fyn, PTP alpha dephosphorylates a distinct inhibitory pTyr residue in Fyn ^52, 53^. Notably, STEP dephosphorylates a pTyr in PTP alpha that normally results in the translocation of PTP alpha to lipid rafts, where it activates Fyn. Thus, STEP has a two-pronged mode of inactivating Fyn: it directly inactivates Fyn and concomitantly prevents activation of Fyn by PTP alpha by blocking its translocation to the membrane.

## STEP and altered synaptic activity in Alzheimer’s disease

**Figure 3.**
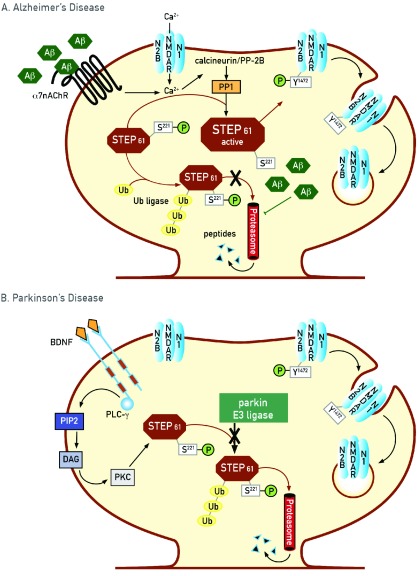
Potential role of striatal-enriched protein tyrosine phosphatase (STEP) in neurodegenerative diseases. **A. Alzheimer’s disease (AD):** Two mechanisms are known to result in an increase in STEP _61_ activity in AD. Beta amyloid (Aβ) binding to the α7 nicotinic acetylcholine receptor (α7nAChR) results in calcium influx and activation of protein phosphatase 2B (PP2B)/protein phosphatase 1 (PP1), which results in dephosphorylation of the regulatory serine ^221^ within the substrate-binding domain of STEP _61_. Dephosphorylation of this site allows STEP _61_ to now associate with and dephosphorylate its substrates. In addition, STEP _61_ is normally ubiquitinated and degraded to remove it from synaptic compartments, as synaptic strengthening requires degradation of STEP _61_. Aβ-mediated inhibition of the proteasome results in a build-up of STEP _61_ levels. The net effect is an increase in STEP _61_ level and activity and the subsequent internalization of synaptic GluN1/GluN2B receptor complexes. For clarity, only one substrate is shown (GluN2B subunit of the N-methyl-D-aspartate receptor [NMDAR] complex), although all will be dephosphorylated by increased STEP activity. Ub, ubiquitin. **B. Parkinson’s disease (PD):** The E3 ligase that leads to the ubiquitination of STEP _61_ is parkin, encoded by the *PARK2* gene. Loss-of-function mutations in *PARK2* are one cause of PD in humans, and STEP _61_ levels are elevated in post mortem samples as well as in animal models of PD. Related to STEP turnover, the growth factor brain-derived neurotrophic factor (BDNF) leads to the activation of protein kinase C (PKC) and the rapid ubiquitination and degradation of STEP _61_. Decreased levels of BDNF may contribute to the pathophysiology of PD, although it remains to be determined whether the decreased BDNF signaling is involved in the increased STEP _61_ observed in PD. DAG, diacylglycerol; PIP2, phosphatidylinositol 4,5-bisphosphate; PLC-γ, phospholipase Cγ; Ub, ubiquitin.

The first suggestion that STEP might contribute to the cognitive deficits in AD came from a study by Snyder and colleagues ^10^, which examined the mechanism by which beta amyloid (Aβ) increases the removal of NMDARs from synaptosomal membranes in rodent neuronal models ( Figure 3). Previous studies suggested that Aβ binds to and stimulates α7 nicotinic acetylcholine receptors (α7nAChRs) ^54– 56^. Aβ binding to these receptors results in calcium influx and activation of a cascade of serine/threonine phosphatases involving protein phosphatase 2B (PP2B; calcineurin) and protein phosphatase 1 (PP1). PP1 dephosphorylates and activates STEP, leading indirectly to the dephosphorylation of GluN2B and GluA2. It was subsequently shown that Aβ inhibits proteasomal activity ^57, 58^, leading to a rapid increase in STEP levels ^9, 59^. Thus, these studies show that both dephosphorylation by PP1 and decreased degradation result in an increase in STEP activity and STEP levels, respectively, and subsequent internalization of GluN1/GluN2B and GluA1/GluA2 receptors ^10, 12, 33, 60^.

Complementing these molecular studies, STEP levels are elevated above normal in the prefrontal cortex and hippocampus of AD patients and in the four AD mouse models tested to date ^9, 33, 61, 62^. It is noteworthy that when STEP knockout mice were crossed with either of two mouse AD models, STEP deficiency restored the expression of NMDARs and AMPARs at the synapse, which was associated with a significant improvement in cognitive function ^33, 60^. In summary, high levels of STEP activity in AD disrupt synaptic activity and the synaptic plasticity required for learning and thereby appear to contribute to the cognitive deficits that characterize early symptoms of this devastating illness.

## STEP and altered synaptic activity in Parkinson’s disease

Parkinson’s disease (PD) is the second most common neurodegenerative disorder after AD and affects millions of people worldwide ^63^. This disorder is characterized by selective loss of dopamine neurons in the substantia nigra and dopamine depletion in the striatum, which eventually lead to characteristic motor abnormalities ^64^. As with AD, there is no cure for PD, only temporary symptomatic relief, highlighting the importance of further research on the molecular basis of these diseases in an effort to develop more effective treatment strategies.

Kurup and colleagues ^65^ recently showed that STEP is upregulated in PD. As discussed earlier under substrates, STEP is normally ubiquitinated and degraded by the proteasome – this process is disrupted in AD ^9^. The more recent study identified parkin as the E3 ligase that ubiquitinates STEP. Deficits in parkin expression, the *PARK2* gene product, are implicated in genetic forms of PD, suggesting the possibility that STEP overexpression might contribute to the etiology of PD. Notably, STEP expression was significantly increased in human sporadic PD post mortem samples ^65^. STEP levels are also increased in animal models of the illness, including Park2 knockout rats, and a toxin-based mouse model. Moreover, increased STEP activity is associated with down-regulation of synaptic proteins in the striatum. Together, these results suggest a convergence of a shared pathway in the regulation of STEP and the etiology of some forms of PD.

## STEP inhibition as a potential treatment for neurocognitive disorders

Cognitive function in AD mice is significantly improved by genetically decreasing STEP activity, as previously discussed ^33, 60^. Such results provide a strong rationale to identify small molecule inhibitors of STEP. Recent studies have led to the isolation of a potent STEP inhibitor, 8-(trifluoromethyl)-1,2,3,4,5-benzopentathiepin-6-amine (known as TC-2153) ^66^. TC-2153 increases the tyrosine phosphorylation of three STEP substrates (ERK2, Pyk2, and GluN2B) in neuronal cultures. Moreover, both 6- and 12-month-old 3xTg-AD mice show significant improvement in cognitive function after TC-2153 systemic injections, where the performance of TC-2153-treated 3xTg-AD mice is indistinguishable from that of wild-type mice in the Morris water maze, Y-maze, and object recognition task. It is important to note that pTau and Aβ levels were unchanged in the 12-month-old AD mice treated with TC-2153 compared to vehicle-treated AD mice, demonstrating that inhibiting STEP activity is sufficient to reverse cognitive deficits without affecting pTau and Aβ levels ^66^.

The specificity of TC-2153 has been examined in several ways, including a comparison of STEP with its closely related PTPs, PTP-STEP-like (PTP-SL) and hematopoietic (He)-PTP. Little difference was evident in the inhibition of truncated versions of the PTPs that contained only the catalytic domain. However, comparative analysis of full-length PTPs suggests a significant degree of specificity for STEP compared to the other PTPs. As mentioned, STEP is present throughout the brain with the exception of the cerebellum, which contains the closely related PTP-SL ^67^. ERK1/2 and Pyk2 are expressed ubiquitously, and multiple PTPs dephosphorylate these proteins in non-brain tissues. However, administration of TC-2153 increases ERK2 and Pyk2 phosphorylation only in the cortex and hippocampus and not in the cerebellum or any of the peripheral organs tested. Moreover, there is no significant increase in ERK2 and Pyk2 phosphorylation over baseline conditions in STEP knockout mice treated with TC-2153 ^66^.

The mechanism by which TC-2153 inhibits STEP activity likely involves the formation of a covalent bond with a cysteine residue within the catalytic domain of STEP ^64^. The oxidative attack and addition of a sulfur to the cysteine promotes a loss of STEP catalytic activity. Mass spectrometry confirmed modifications to the active site cysteine, suggesting that a sulfur from the benzopentathiepin ring is retained. These findings support recent research showing that oxidative regulation of PTPs is an important regulatory mechanism occurring in cells to link tyrosine phosphorylation signaling and redox status ^68, 69^.

## Speculation and future directions for studies of STEP in neurocognitive disorders

STEP levels are clearly elevated in AD and PD. An increase in STEP activity is also observed in mouse models of schizophrenia (SZ) ^70^ (but see 71) and fragile X syndrome (FXS) ^72^. Potential mechanisms for increased STEP activity in these diseases include decreased degradation, evident in AD, PD, and SZ, or an increase in its translation, evident in FXS. Additional, as-yet-unknown mechanisms likely contribute to the regulation of STEP expression and/or activity and thereby contribute to the modulation of synaptic function. In contrast, low STEP activity may contribute to the pathophysiology of other nervous system disorders including alcohol abuse ^73– 75^, stress disorders ^76– 78^, cerebral ischemia ^79^, and Huntington’s chorea ^80, 81^.

Given that both high and low STEP activity contributes to various neuropsychiatric disorders, the original general model whereby STEP suppresses synaptic plasticity requires modification, and it appears to be clear that optimal levels of STEP are required for normal synaptic function. Related to this, a recent study showed that decreased STEP activity in the mouse striatum (through protein kinase A [PKA] phosphorylation of STEP) is important for improving motor learning ^82^. These findings are consistent with earlier studies showing that STEP knockout mice have facilitated hippocampal and amygdala learning but extend the possible involvement of STEP to other types of learning. In addition, it was noted in the Morris water maze paradigm that despite the enhanced learning by STEP knockout mice after the initial training phases, learning to find the location of the platform after it was moved to a new location was impaired ^30^. Thus, when STEP levels are low, extinction may be disrupted because the mice appeared to perseverate on the initial learned task. It will be important for future studies to examine STEP activity in tic disorders, obsessive compulsive disorder, and autism, all disorders characterized by repetitive behaviors as well as difficulties in modulating behaviors in a changing environment.

Recent studies have investigated some of the regulatory mechanisms that promote STEP ubiquitination and degradation. Brain-derived neurotrophic factor (BDNF) and other neurotrophic factors promote the development of synaptic strengthening while STEP opposes it, raising the possibility that they might regulate each other’s activity. It was recently shown that BDNF signaling leads to the rapid ubiquitination and degradation of STEP through TrkB binding and activation of the phospholipase Cγ and protein kinase C (PKC) pathways ^83, 84^. Moreover, decreased neurotrophic factor signaling has been proposed in the pathophysiology of PD ^85– 88^ and, as discussed above, STEP levels are elevated in sporadic PD ^65^. Together, these findings lead to the hypothesis that decreased neurotrophic factor signaling may contribute to the pathophysiology of PD, at least in part, by increasing STEP expression levels. However, further research is necessary to establish a causal relationship between neurotrophic signaling and the increase in STEP levels detected in PD.

Several additional questions raised in this commentary need further study. As mentioned, STEP levels are elevated in a number of CNS disorders that include AD, PD, FXS, and SZ. Reducing STEP activity with genetic or pharmacologic inhibition of STEP reverses the cognitive and behavioral deficits observed in animal models of these disorders. However, it raises the question of how elevated STEP might result in very different types of neurocognitive illness. Presumably, the difference results from brain region- or brain cell type-specific regulation of STEP expression or activity. In support of this hypothesis, STEP is elevated in the striatum in PD, but not in the cortex or hippocampus ^65^, and STEP activity is decreased in alcohol abuse in the dorsomedial striatum but not in the adjacent dorsolateral striatum or nucleus accumbens ^74^. Future studies are needed to address these questions and whether other regulatory mechanisms (e.g. microRNAs) provide differential and region-specific increases, or decreases, in STEP expression patterns.

TC-2153 has been shown to be a useful tool for testing new hypotheses about STEP function in neurocognitive disorders. TC-2153 corrects biochemical abnormalities at the synapse and reverses cognitive and behavioral deficits in mouse models of AD ^66^ and has been used successfully in a number of other cell-based and animal models. TC-2153 is not likely to be useful as a template for further drug development, owing to its chemical properties. However, STEP appears to be an excellent drug target. STEP is brain specific, enriched in frontal brain regions important in cognition, is localized to post-synaptic sites, has limited substrate specificity, and, as an enzyme, has an active site that is amenable to drug development. The rationale for the use of any STEP inhibitor would be to target deficits associated with synaptopathologies found at early stages of neurodegenerative diseases such as AD or PD. STEP inhibition may also be able to complement other therapies that, for example in AD, target the generation or deposition of the Aβ peptide.

A significant focus in studies of STEP has been its ability to dephosphorylate key proteins involved in the regulation of synaptic activity, such as glutamate receptors. It is likely that additional substrates for STEP remain to be identified, some of which may provide a link between the various diseases with which STEP has been associated. Disruption in synaptic connectivity and loss or altered development of dendritic spines have been observed in AD, FXS, and SZ. In this respect, a recent study showed that STEP dephosphorylates SPIN90, a negative regulator of cofilin-mediated actin depolymerization ^89^. When tyrosine-phosphorylated, SPIN90 binds to cofilin, inhibits its activity, and blocks actin depolymerization; this sequence of events prevents the activity-dependent redistribution of key proteins that are required for the morphological changes in synaptic structure that occur during synaptic strengthening. STEP dephosphorylation of SPIN90 reverses this process to effectively promote synaptic reorganization.

A role for STEP in synaptic excitation was observed in several studies showing that a decrease in STEP may increase seizure thresholds. It is noteworthy in this context that STEP deficiency protects hilar interneurons from excitotoxic damage during pilocarpine-induced seizures ^90, 91^. Finally, a recent study showed that STEP plays a role during homeostatic synaptic plasticity through the regulation of AMPAR and NMDAR trafficking ^92^. Further work is needed to confirm and extend these findings.

In summary, the current model of STEP function is that STEP normally opposes the development of synaptic strengthening. Both high and low levels of STEP activity contribute to synaptic dysfunction and to disruptions in behavior and cognitive function. Given the large number of neurocognitive diseases in which STEP has now been implicated, it appears to be a critical nodal point for synaptic regulation. Future efforts should focus on discovering additional disorders in which STEP and synaptic function are disrupted to refine our understanding of how STEP influences neurocognitive function and dysfunction.
